# Suboptimal Clinical Documentation in Young Children with Severe Obesity at Tertiary Care Centers

**DOI:** 10.1155/2016/4068582

**Published:** 2016-09-06

**Authors:** Cassandra C. Brady, Vidhu V. Thaker, Todd Lingren, Jessica G. Woo, Stephanie S. Kennebeck, Bahram Namjou-Khales, Ashton Roach, Jonathan P. Bickel, Nandan Patibandla, Guergana K. Savova, Imre Solti, Ingrid A. Holm, John B. Harley, Isaac S. Kohane, Nancy A. Crimmins

**Affiliations:** ^1^Vanderbilt University School of Medicine, Nashville, TN, USA; ^2^Boston Children's Hospital, Boston, MA, USA; ^3^Harvard Medical School, Boston, MA, USA; ^4^Cincinnati Children's Hospital Medical Center, Cincinnati, OH, USA

## Abstract

*Background and Objectives.* The prevalence of severe obesity in children has doubled in the past decade. The objective of this study is to identify the clinical documentation of obesity in young children with a BMI ≥ 99th percentile at two large tertiary care pediatric hospitals.* Methods.* We used a standardized algorithm utilizing data from electronic health records to identify children with severe early onset obesity (BMI ≥ 99th percentile at age <6 years). We extracted descriptive terms and ICD-9 codes to evaluate documentation of obesity at Boston Children's Hospital and Cincinnati Children's Hospital and Medical Center between 2007 and 2014.* Results.* A total of 9887 visit records of 2588 children with severe early onset obesity were identified. Based on predefined criteria for documentation of obesity, 21.5% of children (13.5% of visits) had positive documentation, which varied by institution. Documentation in children first seen under 2 years of age was lower than in older children (15% versus 26%). Documentation was significantly higher in girls (29% versus 17%, *p* < 0.001), African American children (27% versus 19% in whites, *p* < 0.001), and the obesity focused specialty clinics (70% versus 15% in primary care and 9% in other subspecialty clinics, *p* < 0.001).* Conclusions.* There is significant opportunity for improvement in documentation of obesity in young children, even years after the 2007 AAP guidelines for management of obesity.

## 1. Background

The epidemic of obesity continues unabated. According to the National Health and Nutrition Examination Survey (NHANES) 2011-2012, 8.4% of children aged 2–5 years are obese [1, 2]. Despite reports of recent stabilization of overall obesity prevalence, rates of severe obesity (≥Class 2 obesity, defined as BMI > 120% of 95th percentile) have increased by over 50% since 2000 [3–5], especially in the youngest children [6]. Childhood obesity tends to track into adulthood and portends a dramatic increases in diseases such as atherogenic heart disease, type 2 diabetes mellitus (T2DM), dyslipidemia, sleep apnea, and early mortality [4, 7–9]. Response to medical intervention of obesity is more effective in early childhood compared to adolescents and adults [10, 11]. Hence, prevention and intervention for management of obesity in early childhood are optimal.

In 2007, the American Academy of Pediatrics (AAP) published expert committee recommendations on the prevention, assessment, and treatment of overweight and obese children. The committee emphasized assessment of body mass index (BMI) at every well-child visit as a method of identifying obesity and the first important step in the creation of a chronic care model with community involvement [12]. However, several years after the guidelines, the rates of identification of overweight and obese status in children continue to be low. Prior studies have shown poor documentation of obesity in pediatric patients using ICD-9 codes [13–16]. Few studies have investigated the documentation of the obesity by providers in the progress notes.

Widespread availability of electronic health records (EHRs) and tools such as automatic BMI calculation [17] and alerts for high BMI [18–20] have improved the documentation of obesity in some settings. However, there is limited data on documentation in children under 6 years of age with severe early onset obesity, where the possibility of long-term adverse effects is the highest and early intervention is critical.

This study seeks to identify the rates of clinical documentation of obesity in children under 6 years of age, using the EHRs at two large pediatric academic medical centers. We used structured data such as ICD-9 codes and nonstructured data such as provider notes using natural language processing (NLP) to assess documentation. Based on prior studies, we hypothesized that rates of documentation of obesity in the youngest age group, even among those with severe obesity, would be low.

## 2. Methods

### 2.1. Study Design

This is a retrospective cohort study utilizing EHR data for children with severe obesity between the ages of 1–5.99 years, from both outpatient and inpatient settings at Cincinnati Children's Hospital Medical Center (CCHMC) and Boston Children's Hospital (BCH) from 2007 to 2014. This study was conducted under the auspices of Electronic Medical Records and Genomics (eMERGE) network, a national consortium organized by the National Human Genome Research Institute (NHGRI). Institutional Review Board for research in human subjects approved the protocol at CCHMC and BCH, under a waiver of informed consent.

### 2.2. Data Source

CCHMC has used EpicCare (Epic Systems Corporation, Madison, WI) since 2010 and BCH has used Cerner Solutions (Cerner Corporation, Kansas City, MO) since 2006. Data extraction was performed on the electronic patient records completed during routine clinical care at CCHMC from January 2010 through June 2012 and from January 2007 through November 2014 at BCH. Both inpatient and outpatient records were included.

### 2.3. Algorithm

**Figure 1:**
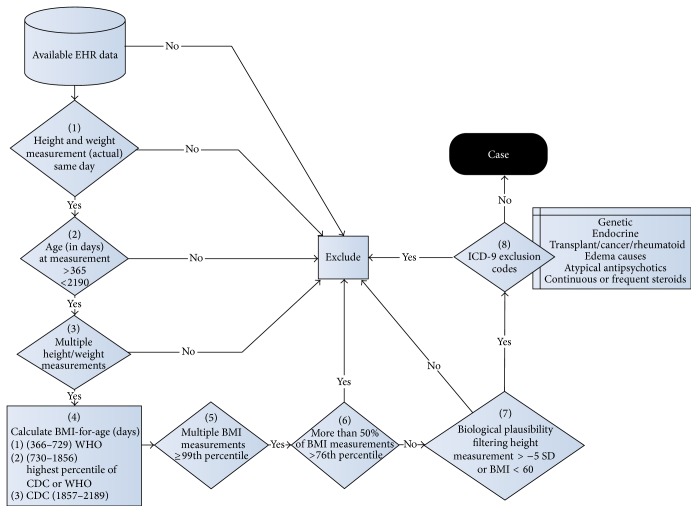
Severe early childhood obesity electronic algorithm. EHR data: available electronic medical record data. Patients required both a height and weight recorded on the same day with height or length >−5 SD for age and gender, at least 2 BMIs ≥ 99th percentile, and >50% of all BMIs > 76th percentile.

As part of the eMERGE project, a validated electronic algorithm was established to identify cases of severe obesity using structured and nonstructured data fields captured in the EHR during clinical care [21]. BMI was calculated by the EHR systems from height (or length, if under the age of 2) and weight data recorded at the same visit by medical assistants and/or nurses during the course of routine clinical care. Age- and gender-specific reference BMI percentiles were automatically calculated within the EHR using Centers for Disease Control and Prevention (CDC) 2000 growth charts for children older than 24 months of age and World Health Organization (WHO) 2006 growth charts for children 12–23 months old. New evidence suggests that BMI is a more useful parameter to measure adiposity in children under 2 years of age, compared to weight-for-length [22]. To minimize biologically implausible values, any height- or length-for-age measurement < −5 SD were removed. Any absolute BMI > 60 kg/m^2^ was also eliminated as biologically implausible for children <6 years of age. A positive case for severe obesity was defined as having a BMI ≥ 99th percentile [12] on two or more different encounters, with more than half of all measurements for that individual with BMI > 75th percentile. If more than one recording was present for a given day, only the first recording for the day was included (Figure 1). Furthermore, if measurements were carried forward across several days of the same inpatient encounter, only the first day of that encounter was included in analysis. Of note, the definition of BMI ≥ 99th percentile for severe obesity was used, as this is readily available at the point of care for the physician. The current EHR systems are not adapted to calculate or display 120% of 95th percentile in keeping with the current definition of severe obesity [23].

Known causes of obesity including endocrine (e.g., Cushing's syndrome), genetic (e.g., Prader-Willi syndrome), malignancy, and connective tissue disorders and diseases causing edema (e.g., renal failure) were excluded using ICD-9 codes and written documentation (Table 1). We excluded these patients because we felt that providers might not document obesity in these patients, not because it was not recognized, but because there were more pressing medical issues to address or because the cause of the obesity was not felt to be endogenous. Prescription data was used to exclude patients on prolonged courses of steroids (longer than 14 consecutive days or three or more separate courses totaling more than 28 days in the six months prior to qualifying weight) or atypical antipsychotics. To validate the algorithm, a manual chart review of 200 charts was performed at each center by at least two physicians (pediatric endocrinology and emergency medicine). A systematic data collection of the chart review was maintained and an interrater reliability > 85%  *F* measure [24] and a positive predictive value of >90% was achieved after the training phase. Any disagreements were adjudicated by discussion.

### 2.4. Documentation of Obesity

Following identification of cases of severe obesity by the algorithm, the available clinical notes and diagnosis codes were extracted for all cases. Natural language processing (NLP) was performed on the clinical notes using a regular expression search with descriptive obesity terms and phrases (Table 2). A broad selection of weight- and obesity-related terms was used to maximize sensitivity. Any encounter that had at least one term from the list in the notes or any of the listed diagnosis codes was considered to have documentation of obesity. Additionally, a notation of an ICD-9 code relevant to obesity in the problem list (Table 2) was considered as positive documentation. An automatic notation of BMI value in the growth chart was not considered documentation, as the EHRs were not configured to provide an alert for a certain percentile of BMI. Individuals were classified as having been “ever documented” if at least one severe obesity encounter showed documentation during the course of care.

### 2.5. Statistical Analysis

Analyses were conducted using SAS version 9.3 (SAS Institute, Cary, NC). Comparisons between centers, or within each center by obesity documentation status, were conducted using Wilcoxon Rank Sum analysis or Fisher's Exact test for continuous or categorical variables, respectively. *p* values <0.05 were considered significant.

## 3. Results

### 3.1. Demographics

We identified a total of 30,463 records for review from CCHMC and BCH of children between the ages of 1–5.99 years that met the inclusion criteria for severe obesity without a pathological etiology. Of these, 891 records were eliminated for biologically implausible values of BMI > 60 kg/m^2^ or height-for-age < −5 SD. Further, 2797 records were eliminated for BMI < 99th percentile. This is in keeping with the 90% PPV of the algorithm. All duplicate records for the same patient at the same encounter were removed, leaving a total of 9887 records of 2588 unique patients. These included 489 unique patients at CCHMC (encounters = 2399) and 2099 at BCH (encounters = 7488).

The median age of patients at their first visit with a BMI ≥ 99th percentile was 34 months (IQR 17, 49), which differed between BCH and CCHMC (BCH 32 months (IQR 17, 48); CCHMC 41 months (IQR 21, 55); *p* < 0.001). Overall, 64% of patients were male (BCH 65%; CCHMC 61%) and were not different by location (*p* = 0.12). Self-reported race and ethnicity varied by the institution, with more multiethnic representation at BCH. Detailed demographic distribution of the cohort is provided in Table 3.

### 3.2. Chart Documentation of Obesity

Using the* a priori* criteria for documentation of obesity, 21.5% of the unique patients (557/2588) had positive documentation of obesity. This rate varied by institution, with 40% ever documented at CCHMC and 17% at BCH (*p* < 0.001). Ever documentation of obesity was lower in those children first seen at <2 years of age (15%) compared to those first seen >2 years of age (26%, Table 4). Consistent with this, those ever being documented had a later age at first BMI ≥ 99th percentile encounter than those never correctly documented, at both centers (both *p* < 0.001), and had more encounters (median 4, IQR 3, 6) compared to those who were not (median 3, IQR 2, 4) (Table 4). The documentation was significantly higher in girls (268/921 = 29% documented) than in boys (289/1667 = 17% documented, *p* < 0.001). African American children were more likely to have a documentation of obesity (115/419 = 27% documented) compared to white children (237/1262 = 19% documented, *p* < 0.001), which was consistent at both institutions (Table 4). Documentation of individual encounters was lower (13.5% overall) than for each individual patient (21.5%, Table 3).

Most of the children with documentation of obesity were Class 2 obesity or higher [25]. At the time of first documentation of obesity (median 48 months, Table 5), the median BMI was 125% of 95th percentile (IQR 116, 140) overall, somewhat lower for BCH (121% of 95th percentile (IQR 114, 133)) than at CCHMC (133% of 95th percentile (IQR 121, 147), *p* < 0.001). Documentation at the first encounter occurred 44% of the time overall and was more common for children ≥2 years old (51%) than children <2 years old (23%, Table 5). There was a median lag overall of 1.5 months (IQR 0, 13) after first encounter to documentation, which was somewhat longer at BCH (5 months (IQR 0, 18)) compared to CCHMC (0 months (IQR 0, 4), Table 5). Approximately half of all visits were documented for each child. The location of the encounter was also a significant factor in determining the documentation of obesity (Table 5). Children seen in the endocrine, nutrition, and obesity clinics had higher rates of documentation 70% of visits overall, compared to primary care (15% of visits) or other subspecialties (9% of visits).

## 4. Discussion

The data from this study identifies poor rates of clinical documentation of severe obesity in young children at two large pediatric academic hospitals utilizing a new, validated EHR algorithm that uses structured and unstructured data (through NLP) to identify chart documentation of obesity. Rates of ever being recognized as obese in young children was 21.5% overall, varying by institution, and much less (13.5%) if evaluated at an encounter level. Most children who were recognized to have some weight issue (based on a broad selection of weight-related terms) were not recognized in the clinical documentation as* obesity* or* severe obesity*, although they would qualify as severe obesity using BMI definition.

Poor documentation of obesity has been shown in previous studies: assessing ICD-9 codes for obesity based on discharge diagnoses identified only 1.7% of children with obesity (mean age for those with obesity not documented, 11.4 ± 4.9 years) [15]. Chart documentation in a general pediatrics clinic showed rates of 34.1% for overweight or obese children of all ages [26], similar to the documentation rates in our study. A national study reviewing documentation and ICD-9 codes in children 2–18 years old showed an 18% rate of identification of obesity [16]. Although these studies investigated identification in all age groups, there was little focus on the youngest age except for mention of poorer rates of documentation. We specifically studied the youngest children with severe obesity to leave out marginal cases and ensure that the degree of obesity was obvious to be acknowledged by the providers. At both institutions, the children who were identified appropriately had a significantly higher BMI than those not documented suggesting that the severity of the obesity played a role in the clinical documentation.

Our study is novel in that it focused on the youngest children. As eating patterns are established early in life, this is a key age to recognize and address obesity. Children who are overweight by kindergarten were found to have four times higher risk of progressing to an obese adolescence [9]. As severe obesity continues into late childhood and adolescence, they develop an increased risk for dyslipidemia, hypertension, and hyperinsulinemia compared to those who are not obese and those with lesser degree of adiposity (BMI between 95 and 99th percentile) [4, 7, 8]. Increasing BMI in children has also been associated with increased risk for other comorbidities [4] and premature death as a young adult [13]. In order to prevent these major complications, obese children must first be identified by medical providers at the youngest age possible for best outcomes.

Similar to previous studies, we identify poor rates of documentation despite evaluating both ICD-9 codes and progress notes. However, we show that the age at first identification of severe obesity was over 36 months at both institutions indicating that children <36 months old were more likely to be missed. Possible explanations for this could be the current lack of clinical guidance for using standardized BMI curves for children less than 24 months or difficulty in approaching this topic at an age where the definitive trajectory of BMI may not yet have been established. It is to be noted that the documentation in children <2 years old was much higher at CCHMC (49% versus 14% at BCH). This perhaps reflects the practice differences due to the presence of a weight management clinic focused on children <6 years old at CCHMC. A lack of clinical focus on endocrine or nutrition issues may account for the gap between these specialty encounters at both sites (BCH: 69%, CCHMC: 72%) and other subspecialty clinics (9% overall). However, even in clinics that should be addressing weight, such as nutrition and endocrine clinics, obesity was not documented at every encounter. The reasons for this are not clear: it could be that weight status was addressed and not documented. Yet, it could be that even these types of providers can be distracted from weight status when addressing non-weight-related chief complaints.

We show that males are less likely to be recognized at both institutions, despite a predominance of males in our study populations. Societal norms perhaps play a role and the younger males are viewed as “stocky” as opposed to obese, which may explain their lack of documentation. Interestingly at both institutions, Caucasians were not documented as often as African Americans. Whether this was due to provider bias is not known but may be potentially explained by the heightened awareness of obesity and its complications in the minority populations [1, 2].

Primary and tertiary medical providers must play an active role in obesity identification at all levels of care in these young children. For children without chronic illnesses, the most frequent encounters with medical professionals occur during the first two years of age. After two years of age, children have less frequent well-child visits. Primary care providers need to pay attention to obesity development in young children even for sick visits, as these visits may represent their only encounters with some families. Although primary care providers are the anchor for obesity management, tertiary care providers and subspecialists can play a valuable role in the identification of obesity, especially those likely to care for adiposity related complications. Common childhood illnesses such as asthma, injuries, and joint pain and abdominal issues can be attributed to obesity even at young ages. Furthermore, obesity in the critically ill patients can lead to acute respiratory distress syndrome, acute kidney injury, and other management difficulties [27–29]. Similarly, there is a higher rate of peri- and postoperative complications in obese individuals [29–31].

The strengths of this study are the use of data from two large tertiary care children's hospitals resulting in a large sample of young children. We were able to utilize NLP with a wide range of terms in addition to ICD-9 codes to assess documentation of obesity with a high sensitivity. Limitations include assessment of the providers' thoughts and actions outside the documented record. The documentation may also have been influenced by the poor reimbursement for the coding of obesity. However, we believe that the documentation of obesity is necessary for ongoing clinical care and should be made regardless of the billing practices. We were not able to obtain data on the clustering of the encounters by provider. It is possible that providers who use the captured methods had more positive records than those who did not. However, the concept of “ever documentation” that credits even a single documentation and analysis at the patient-level overcomes this limitation. Observational study design carries the potential for uncontrolled confounding. However, the large sample size of this specialized age group may overcome this limitation.

## 5. Conclusion

For the first time, this research identifies poor rates of clinically documenting obesity in young children with severe obesity (including those under the age of 2 years) at two large academic children's hospitals utilizing a new, validated EHR algorithm that uses structured and unstructured data to identify appropriate chart documentation. Given the importance of targeting this age group, these results show the need for improvement across all specialties in documentation of severe early onset obesity. With the increasing implementation of health information systems across the country, this offers a useful potential tool to enhance documentation in future, to avoid lifetime complications of excess weight and metabolic complications in the youngest children.

## Figures and Tables

**Table 1:** Diagnoses excluded from electronic health records (EHR) search.

Category of exclusion	Conditions	ICD-9 codes
Endocrine causes	Type 2 diabetes mellitus	250.00, 250.02
	Hypothyroidism	244.9
	Growth hormone deficiency	253.3
	Hypopituitarism	253.2
	Adrenal insufficiency	255.41
	Hypothalamic obesity	259.8
	Precocious puberty	259.1
	Cushing's syndrome	255.0
	Type 1 diabetes mellitus	250.01, 250.03

Genetic causes	Down syndrome	758.0
	Turner syndrome	758.6
	Prader-Willi syndrome	759.81
	Albright hereditary osteodystrophy	756.59
	Bardet-Biedl/ Alström /Noonan/Carpenter's syndromes	759.89

Steroid treatment	Transplant	V42.x
	Rejection	996.8x
	Juvenile rheumatoid arthritis	714.3x
	Inflammatory bowel disease	555.9
	Cancer, brain tumor	191.1, 191.1x, 201.x–208.x
	Histiocytosis	277.89

Edema-causing, gastrointestinal	Congestive heart failure	428.0
	Edema	782.3
	Nephrotic syndrome	581.9
	End-stage renal disease	585.6
	Eosinophilic esophagitis	530.13
	Ulcerative colitis, unspecified	556.9

Psychiatric	Antipsychotic use	—

**(a):** 

Key words/terminology	
Obesity	Obese
Severe obesity	Severely obese
Morbid obesity	Morbidly obese
Central obesity	Overweight
Abnormal weight gain	Excess adiposity
BMI ≥ 85th percentile	High weight
BMI ≥ 95th percentile	Unhealthy weight
BMI ≥ 99th percentile	Excess weight
Elevated body mass index	Elevated weight
Weight too great for length	Chunky
High weight for length	Heavy
	Heavy for age

**(b):** 

ICD-9 coded conditions	
Condition	ICD-9 codes
Obesity, unspecified	278.00
Overweight and obesity	278.0
Morbid obesity	278.01
Overweight	278.02
Localized adiposity	278.1
Overweight, obesity, and other hyperalimentation	278
Abnormal weight gain	783.1
Other unspecified endocrine disorders	259.8

**Table 3:** Description of study populations.

	Overall	BCH	CCHMC	*p* value^*∗*^
(A) Patient-level summary				
*N* unique patients	2588	2099	489	
Age at first 99% percentile visit (months), median [IQR]	34 [17, 49]	32 [17, 48]	41 [21, 55]	<0.001
First 99% percentile visit <2 years (%)	1032 (40%)	892 (43%)	140 (29%)	<0.001
Sex (% male)	1667 (64%)	1367 (65%)	300 (61%)	0.12
Race (%)				
White	1262 (55%)	935 (51%)	327 (67%)	<0.001
Black	419 (18%)	337 (18%)	82 (17%)	
Other	630 (27%)	552 (30%)	78 (16%)	
Unknown	277	275	2	
Ethnicity (%)^*∗*^				
Hispanic/Latino	369 (20%)	331 (25%)	38 (8%)	<0.001
Not Hispanic/Latino	1437 (80%)	988 (75%)	449 (92%)	
Unknown	786	780	6	
Patients ever correctly documented (%)	557 (21.5%)	362 (17%)	195 (40%)	<0.001

(B) Visit-level summary				
*N* visits	9887	7488	2399	
Age at visit (months), median [IQR]	43 [23, 56]	42 [23, 56]	45 [23, 58]	0.05
Sex (% male)	6432 (65%)	4957 (66%)	1475 (61%)	<0.001
Race (%)^*∗*^				
White	5004 (55%)	3329 (50%)	1675 (70%)	<0.001
Black	1523 (17%)	1180 (18%)	343 (14%)	
Other	2513 (28%)	2143 (32%)	370 (15%)	
Unknown	847	836	11	
Ethnicity (%)^*∗*^				
Hispanic/Latino	1437 (20%)	1208 (26%)	229 (10%)	<0.001
Not Hispanic/Latino	5597 (80%)	3435 (74%)	2162 (90%)	
Unknown	2853	2845	8	
Visits correctly documented (%)	1336 (13.5%)	867 (12%)	467 (19%)	<0.001

^*∗*^ *p* value for differences between institutions; individuals with unknown race or ethnicity were not included in *p* value calculation.

*N* (%) or median [IQR] presented.

**Table 4:** Ever documented versus never documented proportions and descriptions by demographic strata.

	Overall		BCH		CCHMC	
	Ever documented	Never documented	Ever documented	Never documented	Ever documented	Never documented
*N* unique patients	557 (21.5%)	2031 (78.5%)	362 (17%)	1737 (83%)	195 (40%)	294 (60%)
Where first visit age <2 years (%)	150 (15%)	882 (85%)	109 (12%)	783 (88%)	41 (29%)	99 (71%)
Where first visit age ≥2 years (%)	407 (26%)	1149 (74%)	253 (21%)	954 (79%)	154 (44%)	195 (56%)
Sex						
Male	289 (17%)	1378 (83%)	188 (14%)	1179 (86%)	101 (34%)	199 (66%)
Female	268 (29%)	653 (71%)	174 (24%)	558 (76%)	94 (50%)	95 (50%)
Race						
White	237 (19%)	1025 (81%)	120 (13%)	815 (87%)	117 (36%)	210 (64%)
Black	115 (27%)	304 (73%)	75 (22%)	262 (78%)	40 (49%)	42 (51%)
Other	157 (25%)	473 (75%)	120 (22%)	432 (78%)	37 (47%)	41 (53%)
Unknown	48 (17%)	229 (83%)	47 (17%)	228 (83%)	1 (50%)	1 (50%)
Ethnicity						
Hispanic/Latino	85 (23%)	284 (77%)	61 (18%)	270 (82%)	24 (63%)	14 (37%)
Not Hispanic/Latino	330 (23%)	1107 (77%)	160 (16%)	828 (84%)	170 (38%)	279 (62%)
Unknown	142 (18%)	640 (82%)	141 (18%)	639 (82%)	1 (50%)	1 (50%)

*Description of ever versus never documented patients*						
*N* visits per person	4 [3, 6]	3 [2, 4]	4 [3, 7]	3 [2, 4]	4 [2, 6]	3 [2, 4]
Age at first visit, months	39 [22, 51]	32 [17, 49]	36 [20, 48]	31 [16, 48]	44 [26, 57]	38 [19, 54]
BMI % of the 95 percentile at first visit	122 [114, 138]	119 [114, 129]	120 [114, 132]	120 [114, 130]	131 [119, 143]	118 [112, 127]

**Table 5:** Characteristics of documentation of obesity.

	Overall	BCH	CCHMC
*N* unique patients	557	362	195
*N* visits	1336	867	467
*N* documented visits per person	2 [1, 3]	2 [1, 3]	2 [1, 3]
Percent of visits correctly documented per person	50 [25, 75]%	50 [25, 75]%	50 [33, 80]%
Patients correctly documented at 1st 99% percentile visit	244 (44%)	130 (36%)	114 (58%)
Where first visit age <2 years	35/150 (23%)	15/109 (14%)	20/41 (49%)
Where first visit age ≥2 years	209/407 (51%)	115/253 (45%)	94/154 (61%)
Age at first documentation, months	48 [35, 60]	48 [35, 60]	48 [35, 59]
Lag to documentation, months	1.5 [0, 13]	5 [0, 18]	0 [0, 4]
BMI % of 95% percentile at first documentation	125 [116, 140]	121 [114, 133]	133 [121, 147]
% visits correctly documented, by clinic^a^			
Primary care	191/1272 (15%)	151/1146 (13%)	40/126 (32%)
Obesity/endocrine/nutrition	665/952 (70%)	505/731 (69%)	160/221 (72%)
Other subspecialty	430/5051 (9%)	195/3584 (5%)	235/1467 (16%)

^a^Total visits with clinic indicated are 5461 for BCH (851 documented) and 1814 for CCHMC (435 documented).

*N* (%) or median [IQR] presented.

## Abbreviations

eMERGE: Electronic Medical Records and Genomics Project.

## Additional Points

*What is Known on This Subject.* Documentation of obesity in children and adults remains low despite availability of automated body mass index (BMI) calculation in electronic health records. Acknowledgment of obesity by providers at an early age is an important first step towards intervention.

*What This Study Adds.* This study demonstrates that children under the age of 6 years are missed in the documentation of obesity. Physicians were found to be more vigilant in documenting obesity in girls and children from minority groups, presenting a significant opportunity for improvement.
